# Antigen Epitope Developed Based on *Acinetobacter baumannii* MacB Protein Can Provide Partial Immune Protection in Mice

**DOI:** 10.1155/2020/1975875

**Published:** 2020-10-20

**Authors:** Xiaojie Song, Guanghui Zhao, Meiling Ding

**Affiliations:** ^1^Department of Respiratory Medicine, Qilu Hospital of Shandong University, Qingdao, China; ^2^Blood Transfusion Department, Qilu Hospital of Shandong University, Qingdao, China

## Abstract

*Acinetobacter baumannii* (*A. baumannii*) is an important opportunistic pathogen widely present in medical environment. Given its complex drug resistance, *A. baumannii* poses a serious threat to the safety of critically ill patients. Given the limited alternative antibiotics, nonantibiotic-based functional anti-*A. baumannii* infection proteins must be developed. In this study, we firstly used a series of biological software to predict potential epitopes in the MacB protein sequence and verified them by antibody recognition and lymphocyte proliferation tests. We finally screened out B cell epitope 2, CD8^+^ T cell epitope 7, and CD4^+^ T cell epitope 11 and connected them to construct a recombinant antigen epitope (RAE). The determination of IgG in the serum of immunised mice and cytokines in the supernatant of lymphocytes showed that the constructed epitope induced an immune response mediated by Th-1 cells. Finally, the challenge experiment of *A. baumannii* infection in mice confirmed that the epitope developed based on MacB, especially RAE, provided incomplete immune protection for mice.

## 1. Introduction

*Acinetobacter baumannii* (*A. baumannii*, Ab) is an important opportunistic pathogen in clinical environment [1]. The bacterium is widely distributed in hospital environments and can survive for a long time in specific departments, such as intensive care units [2], neurosurgery [3], and respiratory medicine [4], easily causing infection in critically ill patients. *A. baumannii* mainly causes respiratory tract infection [5], but it can also cause bacteraemia [6], urinary tract infection [7], secondary meningitis, and surgical site infection [8, 9].

*A. baumannii* is the most common pathogen isolated from patients with hospital-acquired pneumonia in third-grade class-A hospitals in China, and hydrocarbon-resistant strains (CR-Ab) account for 60% to 70% [10]. The drug resistance of *A. baumannii* has become a global challenge [11]; CR-Ab tops the World Health Organization's list of pathogens in need of new antibiotics [12]. In the presence of CR-Ab, when clinicians use high-level antibiotics, such as tetracycline and polymyxin, the treatment effect is occasionally unsatisfactory [13]. This condition also causes heavy financial burden to patients and harmful drug side effects caused. The bacteriostatic or bactericidal strategy based on nonantibiotics is a promising research direction. The outer membrane proteins play an important role in the survival and pathogenesis of *A. baumannii* [14]. MacAB-TolC is an ABC type efflux pump responsible for conferring resistance in bacteria to several antibiotics, while macB was observed to be an essential controlling hub of the network and played a crucial role in MacAB-TolC efflux pump. MacB can act as a potential drug target for successful treatment strategies in *Salmonella enterica serovar Typhi* CT18 [15]. Molecular docking studies of the efflux pump MacB protein revealed that the EPI compound MC207-110 presented a higher binding affinity towards MacB protein. It also can serve as a possible drug target in drug development and discovery [16]. A series of structures of full-length MacB have helped reveal molecular details of its operation. MacB from *A. baumannii* was crystallized in ADP-bound form [17]. This study is aimed at observing the effect of MacB protein on *A. baumannii* by inhibiting its activity.

## 2. Materials and Methods

### 2.1. Bacterial Strains and Mice

*A. baumannii* ATCC 17978 strain was purchased from the American Type Culture Collection (ATCC). Six-week-old female BALB/c mice were purchased from the Experimental Animal Center of Shandong University. All mice were kept in individually ventilated cages. All the animal experiments were approved by the Animal Ethics Committee of Shandong University Qilu Hospital in Qingdao.

### 2.2. Sequence Alignments

The accession number of MacB on the National Center for Biotechnology Information (NCBI) is WP_000165901. Nonredundant protein sequences were selected as the database to run Standard Protein BLAST. Firstly, the range of comparison organisms was limited to *Acinetobacter* (taxid:469). Then, using the same setup, *Acinetobacter* was excluded from the range of comparison organisms. The sequence with the highest score after blasting was saved in FASTA format and transferred to PRALINE server (http://www.ibi.vu.nl/programs/pralinewww/). The results of multiple sequence alignment were manually adjusted for a clear display, base residues that were exactly the same and in the same position were indicated by an asterisk and bases that were not exactly the same were numbered. The higher the score, the more conservative the protein sequence.

### 2.3. Transmembrane Structure and Signal Peptide Prediction

Transmembrane regions are generally rigid structures composed of *α*-helices and should be avoided when designing protein vaccines. TMHMM server (http://www.cbs.dtu.dk/services/TMHMM2.0/) and Octopus server (http://octopus.cbr.su.se/) are classic tools for predicting transmembrane structures. SPLIT (http://split4.pmfst.hr/split/4/) is another website used to predict transmembrane structures. The signal peptide regions predicted by SignalP server (http://www.cbs.dtu.dk/services/SignalP/) were unsuitable as potential antigen epitopes.

### 2.4. Identification of Functional Residues

The ConSurf server (http://consurf.tau.ac.il/2016/) is a bioinformatics tool for estimating the evolutionary conservation of amino acid positions in a protein molecule based on the phylogenetic relations between homologous sequences. Conservation analysis of positions among members from the same family can often reveal the importance of each position for the protein's structure or function. This information is useful for epitope selection in vaccine development.

This server can also predict exposed and buried residues, which are factors to be considered when selecting antigen epitopes, based on the neural network algorithm.

### 2.5. Epitope Prediction

The Immune Epitope Database (IEDB) hosts tools to assist in the prediction and analysis of epitopes, including T and B cell epitopes. Binding to major histocompatibility complex (MHC) is necessary but insufficient for recognition by T cells. The tools provided by IEDB can predict IC50 values (Low IC50 = good binders) for peptides binding to specific MHC molecules. The predicted length of CD8^+^ T cell epitopes bound to MHC class I molecules was set to 9, and that of CD4^+^ T cell epitopes bound to MHC class II molecules was set to 15. The other settings were set in the default mode. Linear B cell epitopes were predicted using Antibody Epitope Prediction server, and BepiPred was selected for prediction. BepiPred predicts the location of linear B cell epitopes using a combination of a hidden Markov model and a propensity scale method. All the selected epitopes were submitted to Sangon Biotech (Shanghai) Co., Ltd for chemical synthesis.

### 2.6. Establishment of *A. baumannii* Infection Model in Mice

Mice were infected with 1 × 10^5^ CFU ATCC 17978 strains by intraperitoneal injection. Mouse serum was collected 7 days later for identification of B cell epitopes, and the spleen was isolated for T cell epitope lymphocyte proliferation test. The B cell epitope with the strongest antiserum recognition ability and two T cell epitopes with the strongest lymphocyte proliferation ability were selected through the above experiments.

### 2.7. Construction of Recombinant Antigen Epitope (RAE)

The three finally screened antigen epitopes were connected into a RAE through the interval sequence Gly-Ser and then delivered to Sangon Biotech (Shanghai) Co., Ltd for chemical synthesis. 2.8. Immunisation of BALB/c mice was randomly divided into five groups, namely, phosphate-buffered saline- (PBS-) negative control group, B cell antigen epitope group, CD8^+^ T cell antigen epitope group, CD4^+^ T cell antigen epitope group, and RAE group, with 15 mice in each group. Each mouse in each group was thoroughly immunised with complete Freund's adjuvant with 200 *μ*g of the target protein. Immunisation was strengthened once on weeks 2, 4, and 6, when it was replaced with incomplete Freund's adjuvant. Serum was collected before each immunisation and on the 14th day after the last immunisation by tail cutting. On the 14th day after the last immunisation, five mice in each group were dissected, and their spleens were isolated for the lymphocyte proliferation test.

### 2.8. Determination of Serum IgG Antibody

Serum IgG, IgG1, and IgG2a were determined by enzyme-linked immunosorbent assay (ELISA) as described previously [18]. Briefly, antigen epitope proteins were coated in a 96-well plate, and after washing and sealing, the serum of each group was added. After washing again, horseradish peroxidase-labelled secondary antibody was added. 3,3′,5,5′-Tetramethylbenzidine was used for colour reaction, and optical density (OD) value was measured at 450 nm by microplating after the reaction was stopped by H_2_SO_4_.

### 2.9. Cytokine Assay

The removed spleen was prepared as a cell suspension, and 5 × 10^5^ cells/well were added to the 96-well plate. Then, the cells were cultured in an incubator at 37°C and 5% CO_2_. Interleukin (IL)-4 was determined by ELISA at 24 h, IL-10 at 72 h, and interferon (IFN)-*γ* at 96 h. ConA was used as the positive control.

### 2.10. Mouse Survival Challenge Experiment

The remaining 10 mice in each group were intraperitoneally injected with 1 × 10^7^ CFU *A. baumannii* ATCC 17978 strain for the attack test. The morbidity and survival time of each mouse were observed and recorded.

## 3. Results

### 3.1. Multiple Sequence Alignment Results

**Figure 1:**
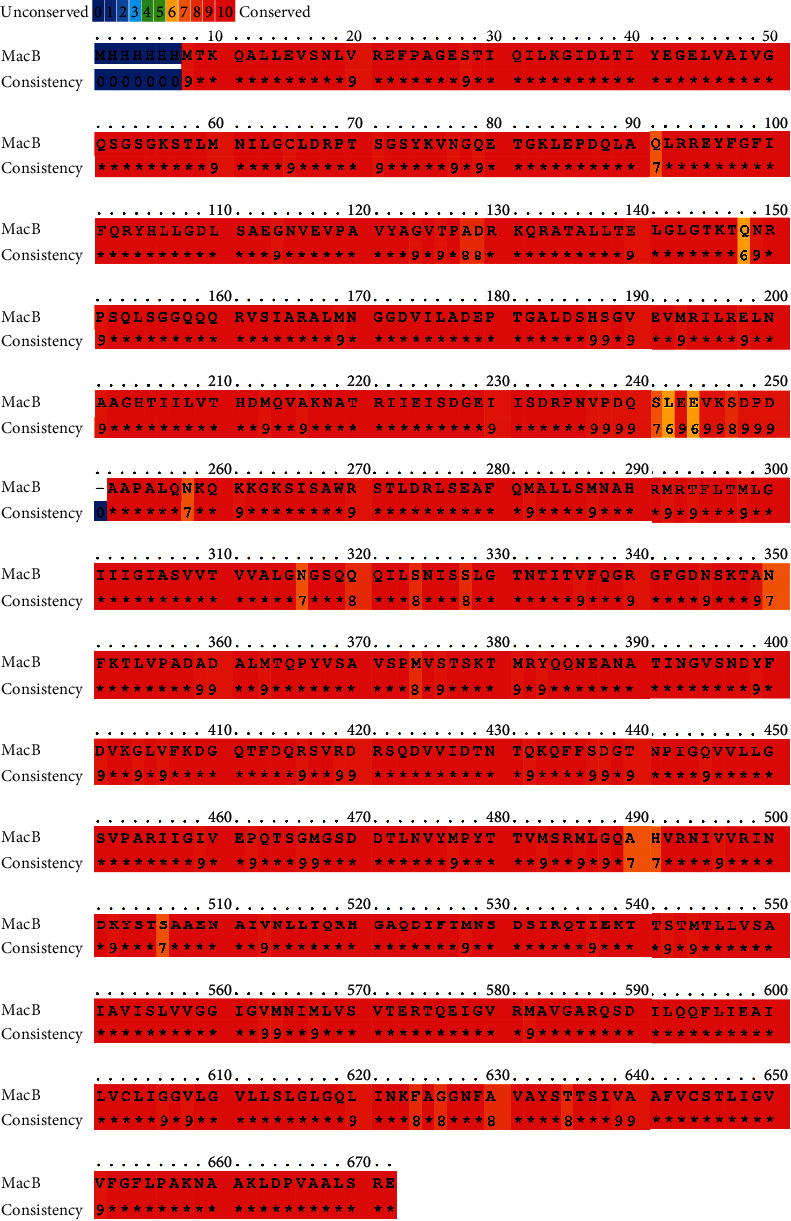
MacB sequence homology with 99 sequences obtained from protein BLAST against *Acinetobacter*. The superposition was performed with the PRALINE programme and adjusted manually. Residue conservation is depicted by blue to red colours.

**Figure 2:**
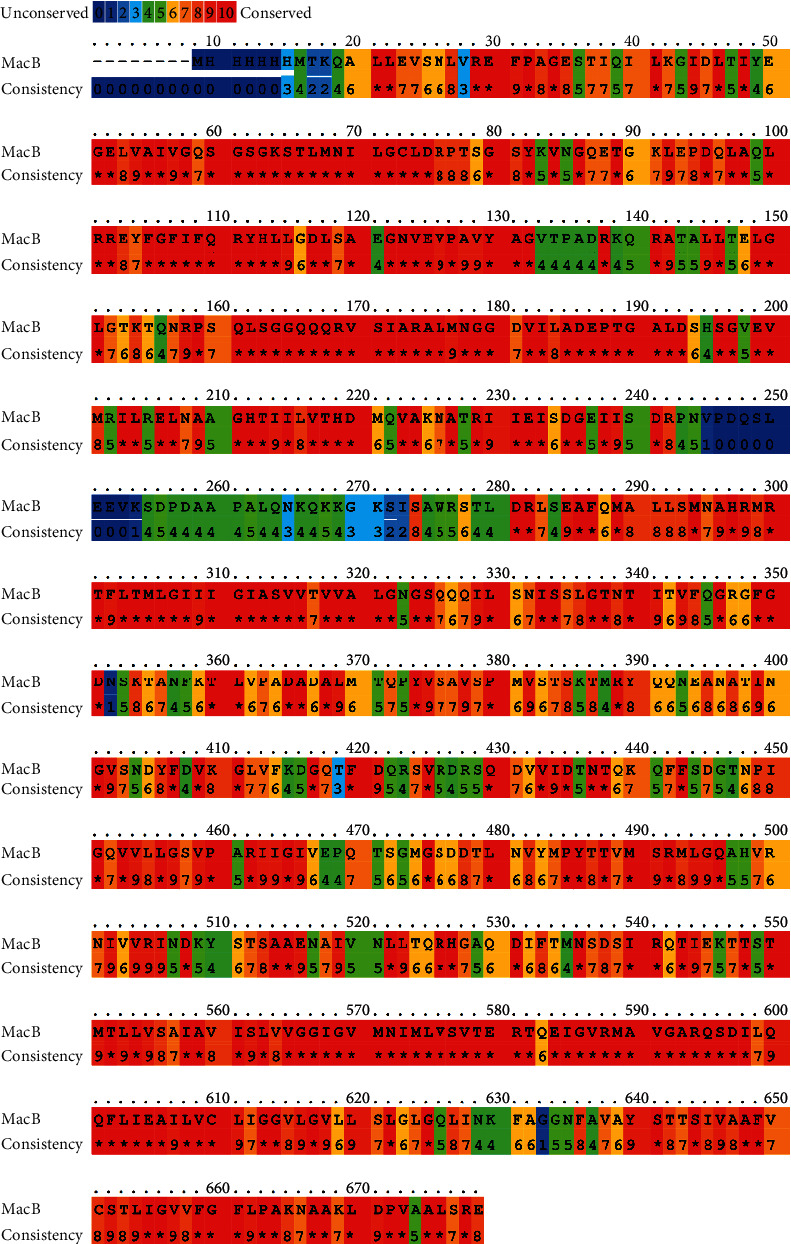
MacB sequence homology with 12 sequences obtained from protein BLAST excluding *Acinetobacter*. The superposition was performed with the PRALINE programme and adjusted manually. Residues conservation is depicted by blue to red colours.

The protein database in NCBI showed that MacB protein contained 664 amino acid residues. Through running protein BLAST analysis, 99 homologous sequences were found in *Acinetobacter* with the defined values of query cover and identity greater than 95% (Figure 1). Using query cover > 95% and identity > 65% as the defined values, protein BLAST analysis was performed in the non-*Acinetobacter* genera. Twelve protein sequences were finally selected, and they are distributed in *Klebsiella pneumoniae*, *Prolinoborus fasciculus*, *Pseudomonadales bacterium*, *Nephila clavipes*, and *Alkanindiges illinoisensis* (Figure 2). When the score of 8 was set as the threshold, MacB was 98.3% conservative in *Acinetobacter* and 61.3% conservative in non-*Acinetobacter*.

### 3.2. Prediction of Transmembrane Structure and Signal Peptides

**Figure 3:**
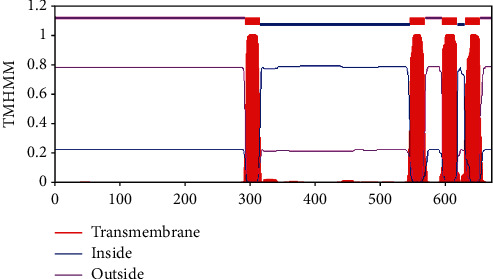
Predicted results of TMHMM server.

**Figure 4:**
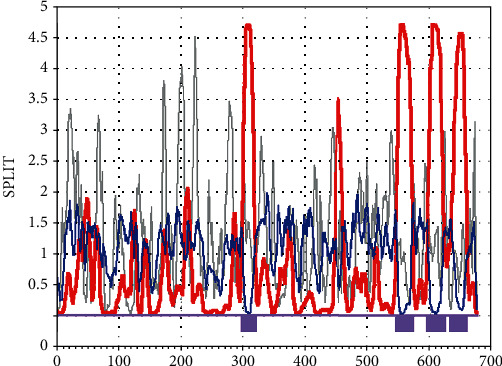
Predicted results of SPLIT server.

**Figure 5:**
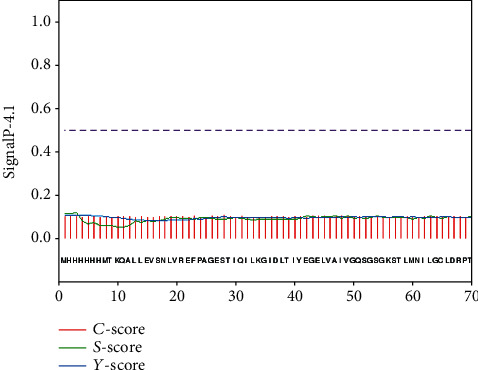
Predicted results of SignalP server.

The prediction results of TMHMM server showed that MacB contained four transmembrane regions, and the prediction results of SPLIT server were consistent with them. These amino acid residues should be avoided during epitope design. Figures 3 and 4 show the detailed results. SignalP 4.1 server predicted that the MacB protein sequence contained no potential signal peptide region (Figure 5).

### 3.3. Prediction of Secondary Structure and Functional Residues

**Figure 6:**
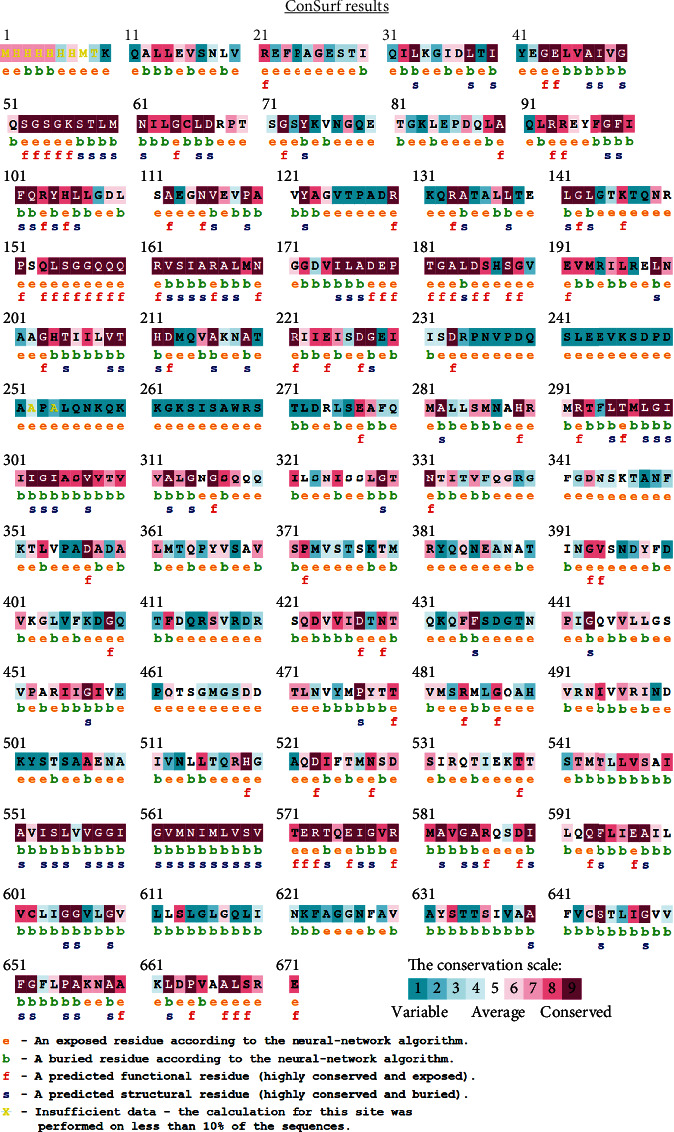
ConSurf prediction of functionally and structurally important residues.

Through the prediction results of ConSurf server, we can screen out the exposed and functional amino acid residues. Only exposed residues can be recognised by corresponding specific antibodies. Functional residues are the key to protein function. The interference of amino acid residue in this region can effectively inhibit its normal function.  Figure 6 shows the detailed prediction results.

### 3.4. Epitope Prediction

**Figure 7:**
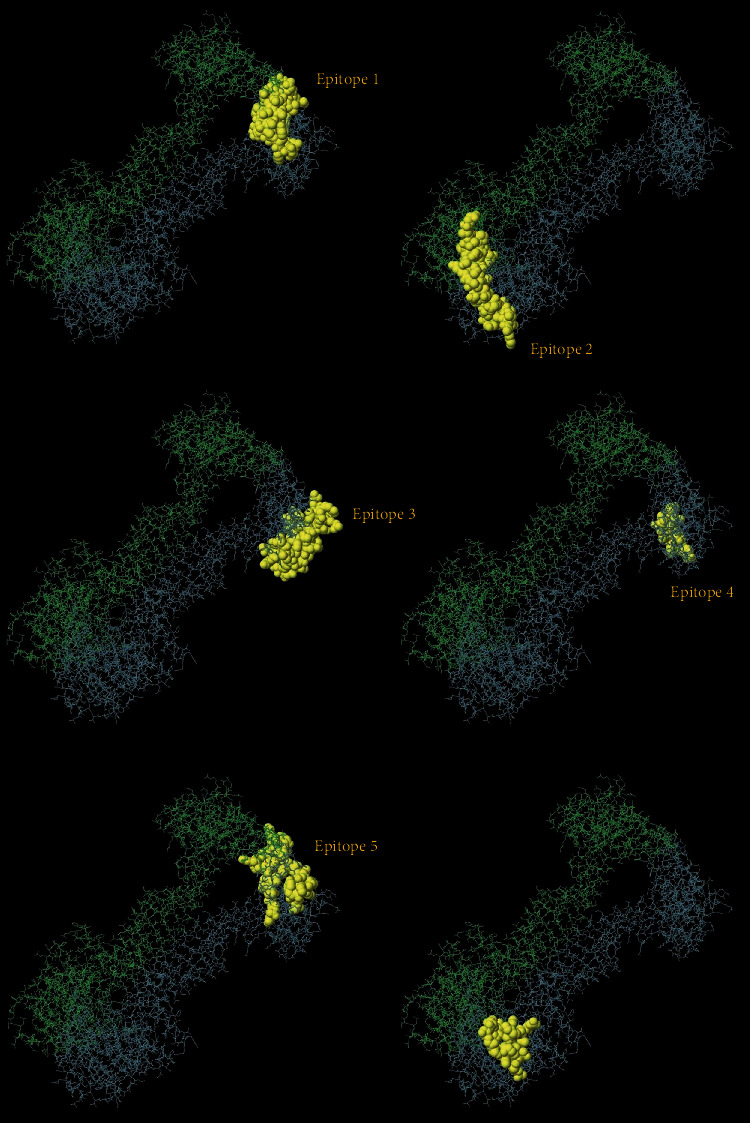
Labelled selected B cell epitopes on the 3D structure. The distribution of potential B cell epitopes on MacB is marked with yellow atmos.

Firstly, the software provided by IEDB was used to predict the B cell, CD8^+^ T cell, and CD4^+^ T cell epitopes. Based on the calculation results of each software, the potential epitopes were preliminarily selected. Combined with the above prediction results, 6 B cell epitopes, 3 CD8^+^ T cell epitopes, and 3 CD4^+^ T cell epitopes were finally selected. The details are shown in Table 1. The six predicted B cell epitopes were displayed in the 3D model of MacB, and the results confirmed that they were all exposed to the surface of the protein structure (Figure 7).

### 3.5. Experimental Verification of Epitopes

**Figure 8:**
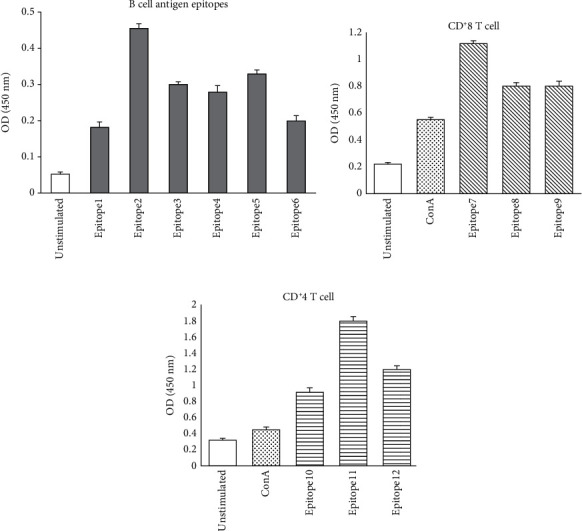
(a) ELISA was used to screen B cell antigen epitopes; (b) lymphocyte proliferation was used to screen CD8^+^ T cell epitopes; (c) lymphocyte proliferation was used to screen CD4^+^ T cell epitopes.

By establishing an animal model of *A. baumannii* infection in mice, serum and spleen cells were collected and isolated, respectively. The result of antibody recognition test shows that epitope 2 has the strongest recognition ability (*P* < 0.05), as shown in Figure 8(a). The results of lymphocyte proliferation assay indicated that epitopes 7 and 11 have the strongest stimulation ability (*P* < 0.05), as shown in Figures 8(b) and 8(c), respectively. After connecting the three epitopes, a recombinant epitope was constructed (KQALLEVSNLVREFPAGESTIQILKGIDLTIYEGE*GS*IEAILVCLI*GS*T QEIGVRMAVGARQS).

### 3.6. Prediction of T Cell Epitopes

T cell epitopes were predicted by IEDB online server, and the predicted results were ranked by percentile. Low percentile represents good binding. Three CD8^+^ T cell and three CD4^+^ T cell epitopes were selected by prediction (Table 1; 35-SYAFDKNQL-43, 175-IAPYLGFGF-183, 139-KRIGNGDTL-147, 118-WAQGLYIAAGAAY LD -132, 119-AQGLYIAAGAAYLDN-133 and 117-PWAQGLYIAAGAAYL-131).

### 3.7. Serum IgG Levels of Immunised Mice

**Figure 9:**
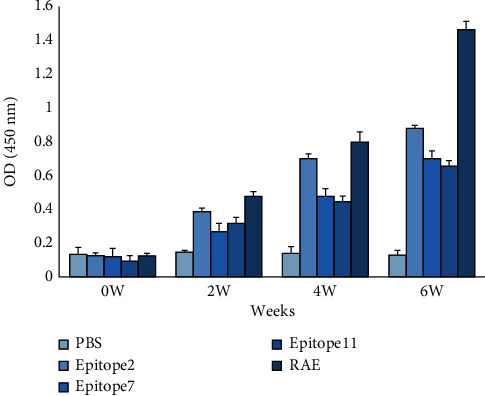
Determination of serum IgG in immunised mice. ^∗^*P* < 0.05.

ELISA results showed that compared with the PBS control group, all epitope proteins can induce mice to produce specific IgG antibody. Among the three single-antigen epitope groups, B cell epitope 2 showed the highest induction ability. The OD value of the RAE group was significantly higher than that of the single-antigen epitope groups, and the difference was statistically significant (*P* < 0.05).  Figure 9 shows the detailed results.

### 3.8. Cytokine Levels

In the measurement of cytokines in the supernatant of mouse spleen cell culture medium on week 2 after the last immunisation, no significant difference was observed in the IFN-*γ* levels between the control and B cell epitope groups. The levels of IFN-*γ* in the two T cell epitope groups and the RAE group increased, and the difference was statistically significant (*P* < 0.05). The RAE group presented the highest OD value. IL-2 and IL-10 showed no significant differences in each group (*P* > 0.05).  Table 2 provides the detailed results.

### 3.9. Mouse Survival Analysis

**Figure 10:**
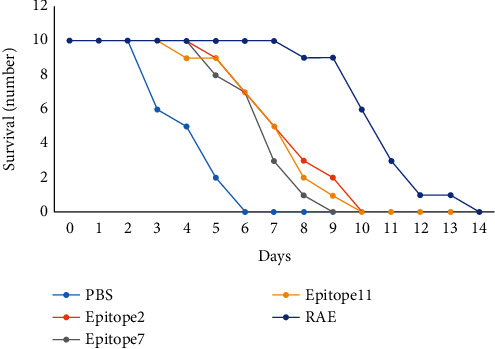
Analysis of mouse survival number.

The challenge test of *A. baumannii* infection was performed on the immunised mice on the second week after the last immunisation. The results showed that the mice in the PBS control group could not tolerate the infection, and all died within 6 days. All the epitope protein-immunised groups showed a certain degree of immune protection, among which the mice in the RAE group died within 14 days, and the survival rate was significantly higher than that of the other groups.  Figure 10 shows the detailed results.

## 4. Discussion

The drug resistance mechanism of *A. baumannii* is complicated, and multiple drug resistance mechanisms, including the production of various *β*-lactamases, decreased membrane permeability, and increased efflux pump expression, often coexist [19]. Therefore, *A. baumannii* can show strong resistance to various kinds of antibiotics commonly used in the clinic. CR-Ab infection severely limits the choice of treatment options for clinicians. For patients with severe CR-Ab infection, a combination of multiple advanced antibiotics is often required, which not only requires a long course of treatment but also results in severe toxicity and side effects of the drug [20]. Thus, multidrug-resistant *A. baumannii* infection poses a huge threat to the lives of patients, especially the critically ill [21].

Given the limited effectiveness of antibiotics, we have committed to develop a bacteriostatic or bactericidal method based on nonantibiotic strategies. Outer membrane proteins play an important role in the survival, drug resistance, and pathogenicity of *A. baumannii* [22]. Deletion or mutation of key outer membrane proteins can seriously affect the survival and pathogenicity of *A. baumannii* [23]. Okada et al. [17] confirmed that the MacA-MacB-TolC tripartite complex is a transmembrane machine and can actively extrude substrates, including macrolide antibiotics, virulence factors, peptides, and cell envelope precursors. These transport activities are driven by ATPase MacB, a member of the ATP-binding cassette superfamily. MacB plays a key role in the material transport of *A. baumannii*. The upregulation of MacB gene expression is closely related to the increased resistance of *A. baumannii*, especially to carbapenem antibiotics and tigecycline [24, 25].

In this study, we firstly analysed the protein sequence conservation of MacB. The results show that MacB is highly conserved within *Acinetobacter* but has low consistency with the protein sequences of other bacterial species, which indicates the feasibility for the development of protein vaccine based on MacB. Then, through a series of bioinformatics software prediction and molecular biology experiment verification, we finally screened out a B cell epitope, a CD8^+^ T cell epitope, and a CD4^+^ T cell epitope and reconstructed them into a complex antigen epitope (RAE). Immunising mice with epitope proteins, especially RAE, can induce the production of a large number of specific IgG and stimulate lymphocytes to produce a high amount of IFN-*γ*. However, the levels of IL-2 and IL-10 showed no increase, suggesting that the antigen epitope protein may stimulate the immune response mediated by Th-1 cells. Finally, the challenge experiments of *A. baumannii* infection in immunised mice confirmed that epitope proteins, especially RAE, provided incomplete immunoprotective effects, based on the fact that it extended the survival time of some infected mice, but ultimately failed to prevent death. The results of this study lay the foundation for the development of functional proteins against *A. baumannii* infection.

## Figures and Tables

**Table 1:** Prediction of epitopes on MacB by bioinformatics.

Rank	Type	Start	End	Sequence	Length	Allele
Epitope 1	B cell	496	530	IVVRINDKYSTSAAENAIVNLLTQRHGAQDIFTMN	35	
Epitope 2	B cell	12	46	KQALLEVSNLVREFPAGESTIQILKGIDLTIYEGE	35	
Epitope 3	B cell	406	460	LVFKDGQTFDQRSVRDRSQDVVIDTNTQKQFFSDGTNPIGQVVLLGSVPARIIGI	55	
Epitope 4	B cell	378	393	TSKTMRYQQNEANATI	16	
Epitope 5	B cell	332	372	TNTITVFQGRGFGDNSKTANFKTLVPADADALMTQPYVSAV	41	
Epitope 6	B cell	69	87	DRPTSGSYKVNGQETGKLE	19	
Epitope 7	CD^+^8 T cell	596	604	IEAILVCLI	9	H-2-Kk
Epitope 8	CD^+^8 T cell	99	107	FIFQRYHLL	9	H-2-Kb
Epitope 9	CD^+^8 T cell	477	485	PYTTVMSRM	9	H-2-Kd
Epitope 10	CD^+^4 T cell	4	18	HHHHMTKQALLEVSN	15	H2-IAd
Epitope 11	CD^+^4 T cell	574	588	TQEIGVRMAVGARQS	15	H2-IAd
Epitope 12	CD^+^4 T cell	577	591	IGVRMAVGARQSDIL	15	H2-IAd

**Table 2:** Cytokine production in cultures of splenocytes from immunised BALB/c mice. ^∗^*P* < 0.05.

Group	Cytokine production (pg/ml)		
	IFN-*γ*	IL-2	IL-10
PBS	56.04 ± 2.11	34.11 ± 1.97	64.32 ± 7.96
Epitope 2	71.33 ± 9.98	45.98 ± 3.28	57.71 ± 8.30
Epitope 7	405.49 ± 29.66^∗^	223.64 ± 21.09^∗^	69.54 ± 11.01
Epitope 11	571.01 ± 38.82^∗^	249.79 ± 34.22^∗^	51.98 ± 6.12
RAE	914.43 ± 48.29^∗^	397.19 ± 23.22^∗^	62.77 ± 9.09

## Data Availability

The data used to support the findings of this study are available from the corresponding author upon request.
